# Dietary Calcium Intake and Calcium Supplementation in Hungarian Patients with Osteoporosis

**DOI:** 10.1155/2013/754328

**Published:** 2013-05-08

**Authors:** Gábor Speer, Pál Szamosujvári, Péter Dombai, Katalin Csóré, Kinga Mikófalvi, Tímea Steindl, Ildikó Streicher, Júlia Tarsoly, Gergely Zajzon, Péter Somogyi, Pál Szamosújvári, Péter Lakatos

**Affiliations:** ^1^Semmelweis University, 1st Department of Medicine, 1083 Budapest, Hungary; ^2^Pálmai-Med Private Medical Praxis, Margit körút 44, 1027 Budapest, Hungary; ^3^Pharma Patent Ltd, 1138 Budapest, Hungary; ^4^Soldra International Ltd, 1024 Budapest, Hungary; ^5^Semmelweis University, Institute of Health Informatics Development and Further Training, 1082 Budapest, Hungary; ^6^National Institute for Sport Medicine, 1123 Budapest, Hungary

## Abstract

*Purpose*. Adequate calcium intake is the basis of osteoporosis therapy—when this proves insufficient, even specific antiosteoporotic agents cannot exert their actions properly. *Methods*. Our representative survey analyzed the dietary intake and supplementation of calcium in 8033 Hungarian female and male (mean age: 68 years) (68.01 (CI95: 67.81–68.21)) patients with osteoporosis. *Results*. Mean intake from dietary sources was 665 ± 7.9 mg (68.01 (CI95: 67.81–68.21)) daily. A significant positive relationship could be detected between total dietary calcium intake and lumbar spine BMD (*P* = 0.045), whereas such correlation could not be demonstrated with femoral *T*-score. Milk consumption positively correlated with femur (*P* = 0.041), but not with lumbar BMD. The ingestion of one liter of milk daily increased the *T*-score by 0.133. Average intake from supplementation was 558 ± 6.2 mg (68.01 (CI95: 67.81–68.21)) daily. The cumulative dose of calcium—from both dietary intake and supplementation—was significantly associated with lumbar (*r* = 0.024, *P* = 0.049), but not with femur BMD (*r* = 0.021, *P* = 0.107). The currently recommended 1000–1500 mg total daily calcium intake was achieved in 34.5% of patients only. It was lower than recommended in 47.8% of the cases and substantially higher in 17.7% of subjects. *Conclusions*. We conclude that calcium intake in Hungarian osteoporotic patients is much lower than the current recommendation, while routinely applied calcium supplementation will result in inappropriately high calcium intake in numerous patients.

## 1. Introduction

As suggested by abundant data, calcium supplementation by itself—although to a modest extent only—can decrease bone loss, and thereby increase bone mineral density (BMD) in osteoporotic patients [1]. According to evidence from the Cochrane database, at least two years of calcium supplementation will result in a BMD increase of 1.66% in the lumbar spine, 1.60% in the hip region, and 1.91% in the distal radius, respectively [2]. However, for the prevention of osteoporotic fractures, calcium supplementation is effective only in combination with vitamin D, although there are data available to refute the importance of this intervention [3]. 

The Decaylos I study, conducted on 3270 elderly French women, aged 84 years on average, evaluated the role of concomitantly administered calcium and vitamin D in reducing fracture risk [4]. The subjects received 1200 mg calcium with 800 IU vitamin D or placebo daily, over three years. By the end of the third year, the number of hip fractures, as well as of all nonvertebral fractures decreased by 29% and 24%, respectively. In a Danish study [5] of 9605 elderly patients, combination therapy with 1000 mg calcium and 800 IU vitamin D daily reduced the number of fractures significantly, by 27%. These findings were confirmed by Tang et al. [6]. In their meta-analysis of 29 trials conducted on 64,000 individuals over the age of 50 years, calcium supplementation was associated with a 12% reduction of the total number of fractures.

Nevertheless, contradicting results have also been reported. In a Dutch study of 2578 women [7], mean dietary calcium intake, which was considered high (868 mg/day on average), was supplemented with 400 IU/day vitamin D for 3.5 years, but even this failed to mitigate fracture risk. In the secondary prevention RECORD study [8], 5292 osteoporotic patients aged over 70 years and with prevalent fractures received 1000 mg calcium with 800 IU vitamin D daily, or placebo. This regimen failed to achieve any significant reduction of fracture risk in the treatment groups. However, only 60% of the subjects were compliant to the therapy (who have taken their study medications according to the intended dosage), which seriously challenges the validity of this finding.

Currently, supplementation with vitamin D and calcium should be regarded as the basic therapy of osteoporosis [9, 10]. In osteoporotic patients, the daily intake of at least 800 to 1000 IU vitamin D and of 1000 to 1500 mg calcium is necessary to increase BMD and to reduce fracture risk [11, 12]. Although calcium and vitamin D are similarly effective whether they are from dietary sources or from supplementation; international and Hungarian data suggest that the intake of both needs an—occasionally substantial—increase [13].

Other potential explanations for the study findings, seemingly challenging the effectiveness of calcium and vitamin D replacement, include the use of nonstandard methods for estimating the daily intake of subjects, and supplementation with various calcium preparations, which produced differing increases of serum calcium levels [14]. Together, these might sometimes have led to suboptimal efficacy of the daily supplementation with calcium and vitamin D. 

The aim of our study was to appraise the dietary intake as well as the supplementation of calcium in osteoporotic patients in Hungary. 

## 2. Material and Methods

### 2.1. Participants and Study Protocol

We conducted a survey using a validated questionnaire (source: modified from Groupe de Recherche et d'Information sur les Ostéoporoses, http://www.grio.org/calcul-apport-calcique-quotidien.php) [15], completed by medical professionals at the osteoporosis centers, following an interview with study subjects. One hundred and eighty-one physicians of 110 study sites, evenly distributed over Hungary, co-operated in this survey. The completed questionnaires accumulated information on osteoporotic 9215 patients. 

The questionnaires were dispensed in batches of fifty, in a form similar to that of copybooks comprising sheets with preprinted serial numbers. Using simple questions (most of which required a numerical entry as a response only), the questionnaire attempted to estimate the intake of calcium-containing foods over a given period (The questionnaire (amended version)). These responses were then used to gauge daily calcium intake based on the frequency of ingestions and of the known calcium content of individual foodstuffs. Additional questions, pertaining to calcium supplementation with medicinal products, served to determine daily intake from this source. The severity of osteoporosis was characterized by bone density measured on the total femur and the lumbar (L_2–4_) spine (BMD was expressed as g/cm² and as *T*-score). Bone mineral density was measured at the total femur and at the lumbar spine by dual X-ray absorptiometry by Lunar Prodigy (GE Medical Systems, Diegem, Belgium), Norland pDEXA (CooperSurgical Inc., Trumbull, CT, USA), and Hologic QDR 4500 (Hologic Inc., Bedford, MA, USA). Coefficient of variation was below 1% at both sites. Osteoporosis was defined according to the WHO criteria, as a *T*-score less than −2.5 at any measured site.

*The questionnaire (amended version)*

                                          Serial N°:

**Ca-intake.hu Study**

version 2.0 of the questionnaire for the appraisal of daily calcium intake in osteoporotic patients

 
**Gender:** female/male
 
**Patient's initials:**
 
**Age:**
 
**Date of birth:**
 
**Body weight:**
 
**Body height:** 
   
*(Please do measure the weight/height of the patient!)*
 
**Lumbar** *T* **-score:**
 
**Region:**L_1-5_/L_2-4_/L_2-5_
 
**Femoral** *T* **-score:**
 
**Densitometer make & model:**
 
**Antiosteoporotic agent:**
   Dosage:   Daily/Weekly/Monthly/Every 3 months/Annually
 
**Vitamin D supplementation: ** tablets/capsules/solution
   Dosage:  x Daily/Weekly/Other
 
**Calcium supplementation:**  Yes/No
   If yes,   tablets.
   Dosage:  x tablets daily.
 
**OTC preparation?**  Yes/No
 
**If POM:**
   Is it prescribed by a specialist?  Yes/No
   Is it prescribed by the family practitioner?  Yes/No
(1) *Do you drink milk every day?*
   If yes, how many glasses (2 deciliters)?
   If not every day, then how many glasses of milk do you drink *per week*?
(2) *Do you eat yogurt, kefir, or curd?*
   If yes, then how many servings of 100 mL do you eat *per week*?
(3) *Do you eat sour cream?*
   If yes, then how many servings of 100 mL do you eat *per week? *
(4) *How much bread do you eat daily?*
   Whole slice: … pieces
(5) *How much pasta do you eat per week?*
   ⋯×100-g servings
(6) *How much meat do you eat per week?*
  - chicken (100-g servings): …/week
  - pork (100-g servings): …/week
  - Parisian/saveloy sausages (100-g servings): …/week
(7) *Do you eat canned fish in oil?*
   If yes, how many cans *per week? *
(8) *How much vegetable do you eat per week?*
   (300-g servings)
  (a) spinach, sorrel: … servings
  (b) green peas, kale, French beans: … servings
(9) *Do you eat cheese regularly?*
   If yes, how many 20-g servings *per week* (see the photo)?
(10) *How much potato do you eat per week?*
   ⋯×100-g servings
(11) *How many eggs do you eat per week?*
(12) *How many medium-sized apples do you eat per week?*
(13) *How many glasses (200-mL) of mineral water do you drink daily?*
  - branded products: Szentkirályi, Natur Aqua
  - branded products: Theodora Quelle, Margitszigeti, Visegrádi
  - other unbranded mineral water:
 
*How long have you been adhering to these eating habits?*
  - all my life
  - for … years
 
*If you have changed your diet long ago, you had done this on:*
  - advice from your doctor
  - other advice

**TO BE COMPLETED BY THE INVESTIGATOR!**

Calcium content of the daily menu:

 (1) …
 (2) …
 (3) …
 (4) …
 (5) …
 (6.a) …
 (6.b) …
 (6.c) …
 (7) …
 (8.a) …
 (8.b) …
 (9) …
 (10) …
 (11) …
 (12) …
 (13.a) …
 (13.b) …
 (13.c) …

**Daily calcium intake:**

Questionnaires from 8033 of the 9215 surveyed osteoporotic patients were complete and thus suitable for evaluation. This means that considering the approximately 330,000 patients (including treated and untreated ones, ESKI, 2009) as reckoned by the collaborating study sites, population coverage exceeded 2.5% altogether. Within the evaluated population (*n* = 8033), the proportion of males was 7.1% (*n* = 569) compared to the dominant 92.9% share of females (*n* = 7464). Mean age of the subjects was 68 years (range 41 to 98). The age distribution of the study population was similar to that exhibited by the national age distribution chart, even for males and females analyzed separately. The body mass index (BMI) was normal in 38.8% of participants (BMI = 18.5-24.9 kg/m^2^); the proportion of underweight subjects was 6.2% (BMI < 18.5 kg/m^2^), whereas 55.0% were overweight (BMI = 25.0–29.9 kg/m^2^) or obese (≥30 kg/m^2^). Mean lumbar and femoral *T*-scores were −2.836 ± 0.03 and −2.434 ± 0.03, respectively. Lumbar and femoral BMD values were related also to age (Table 1).

We did not impose quotas on the selection of responders, in order to accomplish the widest possible population coverage. The course of the survey had two stages, during which a slightly different (amended) version of the same questionnaire was used. Modification was necessary because the original version contained ambiguous questions on cheese consumption. Specifically, 50 grams were defined as a serving, but this proved excessive, resulting in unrealistically high cheese consumption; this was changed to 20 grams in the second version. Additionally, the first version contained two questions on two cheese categories. In the second version, this was reduced to a single, comprehensive question not distinguishing between different types of cheese. Finally, the question on meat consumption reckoned with daily intake in the original version. All these changes had no influence on the standardized analysis of results.

### 2.2. Statistics

The initial step of data processing consisted of digitizing the questionnaires, performing the digitally assisted recording of data in duplicate, and finally, correcting errors. Handwritten notes modifying the frequency entries made on the questionnaires were also recorded (e.g., when the investigator invalidated the “daily” specification by striking it through and entered “weekly” in replacement before completing the entry on bread consumption). Error correction involved checking for records containing logical inconsistencies, outlier values, and skipped entries. Questionnaires containing uncorrectable errors were excluded from the analysis along with those that were deemed unusable for other reasons. The latter included duplicates (identified by matching patient's initials, date of birth, and gender on questionnaires from the same or from a nearby study site); sheets without the age or gender of the subject (if retrieving these data was not feasible, for example, owing to the absence of the date of birth or the information on marital status). Sheets containing data from subjects without osteoporosis (as evidenced by *T*-scores of the lumbar spine and of the femur) were also discarded. As no selection quotas were imposed during the survey, weighing was used to ensure the representativeness of the sample. This was necessary, because the proportions of patients included in the individual counties were at variance with nationwide data on patient distribution. For example, the proportion of patient data accumulated from Tolna County was larger than that obtained from Vas County, whereas national data show a different distribution. Therefore, the results from both counties were corrected with an appropriate multiplier—using a method known as element number stabilization weighing, to obtain results matching their nationwide weights. We recruited the entire Hungarian population of osteoporotic patients as reference (data source: National Institute for Quality and Organizational Development in Healthcare and Medicine—Informatics and System Analysis Directorate/ESKI/, 2009). It is defined as the total number of individuals (specified according to county, age, and gender) receiving in-/outpatient care within the Hungarian health care system, as well as any form of osteoporosis is specified in their primary care reports as an underlying or accompanying disorder. Patients were reckoned by using the data on their age and residence (by county), as recorded at the first presentation during the actual year. The numbers of patients per counties and male-to-female ratios were approximated to the national average. As—among others—age was taken into account during weighing, this method we consider element number stabilization weighing for three dimensions (age, gender, and region).

During the calculations, in the knowledge of the calcium content of selected foodstuffs, we performed the multiplication corresponding with the given frequency period to obtain per patient calcium intake in total and for each foodstuff. Data accumulated with the two versions of the questionnaire were pooled, in order to increase sample size. As regards to the original version of the questionnaire, calcium intake calculated from responses to the question on cheese consumption was 2.5 times higher than that reckoned from the entries of the corresponding item of the amended version. Considering the amendment of the original version after the preliminary analysis (changes included attaching a photo make estimating the quantity of consumed cheese easier), we implemented the following correction. Calcium intake from cheese consumption, as estimated with the original version, was adjusted with the quotient of the means determined with both versions.

These data on dietary calcium intake were used to exclude unrealistically low or high values, and the same procedure was then performed on calcium supplementation data. Disease severity and the appropriateness and efficacy of the diet and medication were compared with each other, as well as by regions. No sample size calculation was performed prior to conduct of the study. For the data analysis, we performed ANOVA tests and linear regression models, respectively. The significance level (alpha) was used 0.05. The given values at the numeric results are the 95% confidence intervals (CI) of the results. The analyses were performed using the SPSS (SPSS Inc., Chicago, IL, USA) software package (IBM SPSS Statistics 20.0.0).

## 3. Results

Of the 8033 evaluated osteoporotic subjects, 5733 (71.7%) received vitamin D supplementation with a mean dose of 756 ± 8.8 IU (68.01 [CI95: 67.81–68.21]), which is essentially in agreement with the recommendations of current professional protocols. Hydroxylated vitamin D was administered to 13.3% of patients. We did not detect any relationship between vitamin D supplementation and BMD values.

**Figure 1:**
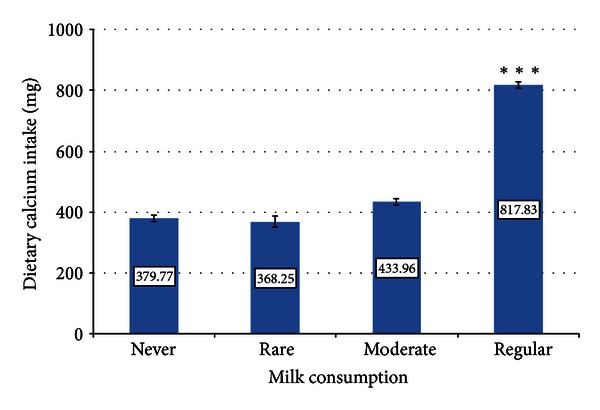
The relationship between the volume of milk consumption and daily calcium intake (*P* < 0.001): the higher was the daily milk consumption, the greater became dietary calcium intake. *Note*. Low milk consumption: never or less than a glass (two deciliters) of milk per week (rare); moderate milk consumption: less than half a glass (one deciliter) of milk a day; regular milk drinkers: more than half a glass (one deciliter) of milk daily.^∗∗∗^ *P* < 0.001 versus abstainers and subjects with low or moderate milk consumption.

The mean dietary calcium intake was 665 ± 7.9 mg (68.01 [CI95: 67.81–68.21]). It was influenced by several factors. These include gender, as the daily intake was significantly lower in males than in females −599 ± 22.7 versus 673 ± 8.4 mg (68.01 [CI95: 67.81–68.21]) (*P* < 0.001). Additional influencing factors included the presence as well as the volume of milk consumption (Figure 1). The higher was the daily milk consumption, the greater became dietary calcium intake. Using this parameter, we assigned the study population into the following four subsets: (1) patients abstaining from milk, (2) subjects with low consumption (less than one glass—200 mL—of milk per week), (3) moderate consumption (less than a half glass—100 mL—of milk a day), or (4) regular milk drinkers (more than a half glass—100 mL—of milk daily). The proportions of subjects in these categories were 20.1%, 3.8%, 12.5%, and 63.6%, respectively. According to this stratification, dietary calcium intake was significantly (*P* < 0.001) greater in regular milk drinkers than in other subjects. The daily intake of calcium from dietary source was not related to BMI.

**Figure 2:**
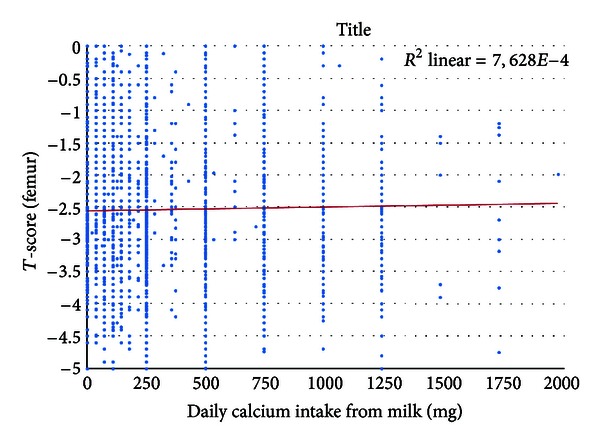
The relationship between the presence of milk consumption and the *T*-score of the femur (*P* = 0.041).

*T*-scores correlated with dietary calcium intake, according to a multiple relationship. The quantity of calcium from food sources was not related to the BMD of the femur (*P* > 0.1), whereas a positive correlation could be demonstrated with lumbar *T*-score (*P* = 0.045). Analyzing BMD exclusively from the aspect of milk consumption, a positive correlation could be shown with femoral BMD, but not for lumbar BMD (Figure 2). In other words, a daily milk consumption of one liter (providing 1200 mg calcium) increased the *T*-score of the total femur by 0.133.

Of the 8033 subjects, 5813 received some form of calcium supplementation, which proved adequate for 72.6%. The mean daily dose of the supplemented calcium was 558 ± 6.2 mg. Analysis by age did not reveal any significant difference, whereas comparison by gender did: it was on average 560 ± 6.6 mg (68.01 (CI95: 67.81–68.21)) in females and significantly smaller 534 ± 18.9 mg (68.01 (CI95: 67.81–68.21)) in males (*P* = 0.012). 

The cumulative dose of calcium from dietary intake and from supplementation was significantly related to lumbar BMD (*r* = 0.024, *P* = 0.049), while no relationship could be detected with femoral BMD (*r* = 0.021, *P* = 0.107).

**Figure 3:**
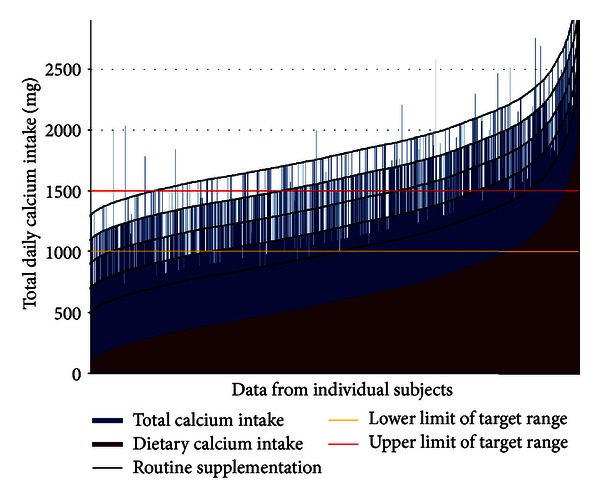
The relationship between calcium intake from supplementation and from dietary sources. *Note*. The recommended RDA of 1000 to 1500 mg total calcium intake is met in approximately 34.5% of patients only. In the majority of subjects, total daily calcium intake is either below or above the recommended range.

Figure 3 illustrates calcium supplementation in relation to dietary calcium intake. The recommended daily allowance of 1000 to 1500 mg calcium was fulfilled in as low as 34.5% of treated patients only. In the majority of osteoporotic patients, total daily calcium intake was below this target or exceeded it substantially.

## 4. Discussion

The Ca-Intake.hu study was the first Hungarian project to evaluate daily calcium intake in osteoporotic patients. Earlier, only Biró et al. have published data on Hungarian healthy adolescents [16]. According to their findings, daily calcium intake is as low as 800 mg in boys and 700 mg in girls. In France, for example, it is 754 mg—that is, half of the recommended RDA—among urban dweller women with postmenopausal osteoporosis [17]. Our analysis of dietary calcium intake in more than 8000 patients, which is an outstandingly large population even in international comparison, has supplied evidence confirming that calcium intake among osteoporotic patients in Hungary is even lower than most of the published data.

Additionally, the Ca-Intake.hu study supplied information on average daily calcium intake from supplements, which was 558 mg. In view of the dietary intake, this amount of calcium results in an adequate total calcium intake in the lesser proportion (34.5%) of patients only, and it is below or well above the recommended in the majority of osteoporotic patients. This finding is probably a consequence of routinely handled calcium supplementation doses. This practice can be seen even in clinical pharmacology trials conducted in osteoporosis. The study protocol only rarely prescribes the appraisal of calcium intake along with the consequential adjustment of supplementation. Not withstanding the conflicting data, 1000 to 1500 mg/day calcium and 800 to 1000 IU vitamin D in combination are likely to increase bone mass and mitigate fracture risk effectively [18–21]. Actually, suboptimal dosing with calcium could be a potential explanation for the findings from the international studies that failed to demonstrate the beneficial effect of calcium and vitamin D supplementation either on bone mass or on of fracture prevention. In the RECORD study [8], the unfavorable results are attributable to poor patient compliance; however, the under-dosing of calcium supplementation is just as deleterious. Moreover, it should be kept in mind that excessive calcium intake can increase the number of bone fractures [22] as well as the incidence of prostate cancer in males [23].

In our study, milk consumption correlated with BMD of the total femur, whereas calcium from other foods or total daily calcium intake had no such relationship. The opposite holds true for the bone density of lumbar vertebrae which was not related to milk consumption but showed a positive correlation with the cumulative dose of calcium from dietary intake and from supplementation. Others reported contrary findings in adolescents: bone densitometry of the lumbar spine (without appraisal of the femur) showed a significant relationship (mediated by insulin-like growth factor 1/IGF-1/) between BMD and milk consumption, but not with calcium from other dietary sources [24]. Potential explanations for these findings include the concomitant intake of additional nutrients (such as magnesium) and proteins present in milk. IGF-1, as well as milk proteins have been shown to exert an anabolic effect on bone. IGF-1 increases the number and enhances the activity of osteoblasts, while stimulating collagen synthesis by these cells [25]. The beneficial effect of milk consumption as well as of treatment with the extract of the milk basic protein fraction on bone turnover (both in cortical and in trabecular substance) in adolescents as well as in adult or elderly women have been reported by others [24, 26, 27]—although contradicting data also exist [28]. The STRAMBO study, for example, showed in males under the age of 65 a harmful effect of a daily calcium intake less than 600 mg both on cortical and on trabecular bone [29].

Our study also has several limitations. The study results inherited a limitation from the questionnaire distribution. At the beginning of the study, no sample size calculation was made, so this resulted in an unbalanced distribution of patient numbers from each Hungarian region. Though these limitations apply, the relatively high sample size strengthens the power of the study. The questionnaire design itself leads to concerns about our results. Data entry and cleaning efforts had been made to identify and clean the strike throughs of weekly/daily frequencies. The one-time questionnaire method should be replaced by digital questionnaire or a week-long food and drink logging in the future to avoid biases resulting from the patient's situation being interviewed personally by their physician. Another limitation is caused by the exact definition of food quantities for that we have to define more robust method to ensure that all patients understand the objective food amounts. Strait methods of measuring calcium intake via water and other drinks should also be determined for the future. In conclusion, these limitations do not affect our main finding about the inappropriate methodology in the field of calcium supplementation.

As confirmed by the Ca-Intake.hu study, the bone density of lumbar vertebrae positively correlated with the total daily intake of calcium from food and from supplementation. Boonen et al. [18] processed the results of six randomized clinical studies involving 45,000 patients and found an 18% reduction of hip fractures with calcium and vitamin D supplementation. The ICARO study [30] was the first to demonstrate that the lack of compliance with calcium and vitamin D regimens reduces the antifracture efficacy of antiosteoporotic therapies, compared to that seen in controlled studies (and in combination with adequate calcium and vitamin D supplementation). Thus, any of the tested antiosteoporotic agents can achieve its maximum effect only in the presence of calcium and vitamin D sufficiency. According to the findings of the Ca-Intake.hu study, the level of vitamin D supplementation in Hungary is on par with that recommended by current international guidelines.

## 5. Conclusion

In conclusion, calcium intake in Hungarian osteoporotic patients is much lower than the current recommendation, while routinely applied calcium supplementation will result in inappropriately high calcium intake in numerous patients.

## Figures and Tables

**Table 1:** The baseline characteristics of our patients.

Gender (%)	Male = 569 (7.1) Female = 7464 (92.9) Total = 8033 (100)
Age (years)	41–98 Mean: 68.01
Lumbar *T*-score	−9.37–1.5 Mean: −2.836 ± 0.03
Femoral *T*-score	−8.66–0.9 Mean: −2.434 ± 0.03
Weight (kg)	32–130 Mean: 66.78
Height (cm)	120–194 Mean: 159.93
BMI (kg/m^2^)	13.62–60.26 Mean: 26.11
Vitamin D supplementation (%)	*n* = 5773 (71.7)
Calcium supplementation (%)	*n* = 5813 (72.6)
Mean dietary calcium intake (mg)	665 ± 7.9 Female = 599 ± 22.7 Male = 673 ± 8.4
